# Epitope-Based Vaccines against the *Chlamydia trachomatis* Major Outer Membrane Protein Variable Domain 4 Elicit Protection in Mice

**DOI:** 10.3390/vaccines10060875

**Published:** 2022-05-30

**Authors:** Amanda L. Collar, Alexandria C. Linville, Susan B. Core, Kathryn M. Frietze

**Affiliations:** 1 Department of Molecular Genetics and Microbiology, University of New Mexico Health Sciences, Albuquerque, NM 87131, USA; collaral@salud.unm.edu (A.L.C.); alexandria.linville@huskers.unl.edu (A.C.L.); score@salud.unm.edu (S.B.C.); 2 Clinical and Translational Science Center, University of New Mexico Health Sciences, Albuquerque, NM 87131, USA

**Keywords:** *Chlamydia trachomatis*, vaccine, virus-like particle, antibodies, epitope, bacteriophage, affinity selection

## Abstract

*Chlamydia trachomatis* (Ct) is the most common bacterial sexual transmitted pathogen, yet a vaccine is not currently available. Here, we used the immunogenic bacteriophage MS2 virus-like particle (VLP) technology to engineer vaccines against the Ct major outer membrane protein variable domain 4 (MOMP-VD4), which contains a conserved neutralizing epitope (TTLNPTIAG). A previously described monoclonal antibody to the MOMP-VD4 (E4 mAb) is capable of neutralizing all urogenital Ct serovars and binds this core epitope, as well as several non-contiguous amino acids. This suggests that this core epitope may require conformational context in order to elicit neutralizing antibodies to Ct. In order to identify immunogens that could elicit neutralizing antibodies to the TTLNPTIAG epitope, we used two approaches. First, we used affinity selection with a bacteriophage MS2-VLP library displaying random peptides in a constrained, surface-exposed loop to identify potential E4 mAb mimotopes. After four rounds of affinity selection, we identified a VLP-displayed peptide (HMVGSTKWTN) that could bind to the E4 mAb and elicited serum IgG that bound weakly to Ct elementary bodies by ELISA. Second, two versions of the core conserved TTLNPTIAG epitope (TTLNPTIAG and TTLNPTIAGA) were recombinantly expressed on the coat protein of the MS2 VLP in a constrained, surface-exposed loop. Mouse immune sera IgG bound to Ct elementary bodies by ELISA. Immunization with these MS2 VLPs provided protection from vaginal *Chlamydia* infection in a murine challenge model. These data suggest that short peptide epitopes targeting the MOMP-VD4 could be appropriate for Ct vaccine design when displayed on an immunogenic bacteriophage VLP vaccine platform.

## 1. Introduction

*Chlamydia trachomatis* (Ct) is the cause of the most common bacterial sexually transmitted infection. The World Health Organization has identified sexually transmitted Ct as a priority for vaccine development due to the high cost of treatment and screening, as well as the long-term sequelae associated with infection in women [1,2,3]. Ct infections are caused by multiple distinct circulating urogenital serovars. However, eliciting long-lasting, protective immunity against all Ct urogenital serovars has proven challenging because protective epitopes tend to be variable [4,5].

Ct serovar identity is determined by variation in the amino-acid sequence of the major outer membrane protein (MOMP) which is present on the surface of Ct elementary bodies (EBs)—the infectious form of *Chlamydia*. MOMP contains four variable domains and five constant domains [4,5]. A highly conserved epitope present in the variable domain 4 (VD4) region of MOMP has become an attractive vaccine target and was included as part of a Ct vaccine recently tested in a Phase I clinical trial [6,7,8]. This conserved epitope is present across all Ct serovars, as well as other human pathogenic *Chlamydia* species, suggesting an important biological role for this epitope in the function of MOMP. Additionally, several monoclonal antibodies that neutralize multiple Ct serovars have been identified that recognize this conserved VD4 epitope [9,10].

Since MOMP is an important mediator of host cell attachment and entry and is an immunodominant antigen, many previous Ct vaccine efforts have targeted MOMP, attempting to isolate and generate the protein in large quantities [11,12,13,14,15,16,17,18,19]. One such strategy involved isolating recombinant or denatured MOMP from *C. muridarum* (Cm, a related *Chlamydia* species that infects mice and is frequently used as a surrogate model for vaccine testing) and using this preparation as an immunogen [15,17,18]. While mice in these studies immunized with MOMP recombinant proteins exhibited some immune response (specifically, the presence of MOMP-specific antibodies), no significant differences in vaginal shedding were observed between the groups immunized with the recombinant MOMP proteins and negative control groups immunized with ovalbumin following vaginal challenge with Cm. Conversely, native MOMP, when isolated directly from mammalian cells infected with Cm in such a way that the MOMP retains its native structure, elicits a robust humoral and cell-mediated immune response that is capable of protecting against infectivity and infertility in mice [20]. Unfortunately, native MOMP extraction from Ct is both laborious and time-consuming to complete on a large scale, making this strategy unfeasible for translation to human vaccination trials [16]. These previous studies suggest that conformation of key epitopes may be critical for eliciting protective immunity.

Recently, a promising vaccine candidate has emerged, consisting of a recombinant engineered multivalent vaccine construct based on T- and B-cell epitopes from variable domain 4 (extVD4) regions from the most prevalent serovars of Ct (Svs) D, E, F, and G, coupled with CAF01 adjuvant [6,8]. This vaccine contains key neutralizing epitopes in the VD4 domain with additional conserved T-cell epitopes [6,7]. This vaccine elicits cross-reactive antibodies toward multiple Ct serotypes in vitro, and half of naïve mice given sera from vaccinated mice exhibited protection against a primary Ct vaginal challenge [6]. This study confirmed that antibodies on their own, elicited by the vaccine that targets the VD4 domain, can prevent the establishment of Ct infection in Rag1 knockout mice deficient in mature T and B cells [6]. Additionally, studies of the structure of the antigen included in this vaccine indicate a complex but regular structure (again pointing to the possibility that conformation may be important for protection elicited by MOMP antigens.

We hypothesized that an MS2 bacteriophage virus-like particle (VLP) vaccine platform might provide an alternative approach for eliciting protective antibodies targeting MOMP-VD4. MS2 bacteriophage VLPs are an attractive vaccine platform, as they can elicit high-titer, long-lasting antibodies to foreign peptides displayed on their surface, particularly short peptides that are normally not immunogenic [21,22,23,24,25,26]. MS2 VLPs, in particular, are amendable to the display of short peptide epitopes in a constrained surface-exposed loop, mimicking conformational epitopes that naturally form loop structures [21,24,27]. Furthermore, MS2 VLPs can be used in a novel affinity selection platform called Deep Sequence-Coupled Biopanning [23,28,29,30,31]. Using this technology, we can identify conformational mimics that bind to a specific monoclonal antibody [23]. This is achieved by creating large libraries of MS2 VLPs that display short random peptide sequences in a surface-exposed beta-hairpin structure called the AB loop. Because individual members of the VLP library encapsidate their own coding mRNA, VLPs with specific binding characteristics can be selected from the library, and specific binding sequences can be identified by utilizing RT-PCR and deep sequencing [28,29,30,31,32]. This technology has previously been used to identify conformational mimics of mAbs to *Plasmodium falciparum* [23] and *Staphylococcus aureus* [33].

Here, we report our efforts to engineer vaccines that elicit protective antibodies to the conserved VD4 epitope of Ct. We hypothesized that the conformational context of the conserved VD4 MOMP epitope could be important for the functionality of antibodies. With this in mind, we used two approaches. First, utilizing affinity selection, we used murine monoclonal antibody, E4, which neutralizes all urogenital Ct serovars and binds to the VD4 epitope, to pan a library of MS2 VLPs to identify potential conformational mimics that could be utilized as vaccine candidates. Second, we engineered recombinant MS2 VLPs displaying versions of the core conserved VD4 epitope (TTLNPTIAG) in the AB loop of the MS2 VLPs via site-directed mutagenesis. These vaccines were tested for efficacy against *Chlamydia muridarum* in a murine vaginal infection model.

## 2. Materials and Methods

### 2.1. Affinity Selection of Random Peptide MS2-VLP Library with E4 mAb

E4 mAb was a kind gift from Dr. Luis de la Maza at the University of California-Irvine [10]. Using Protein G Dynabeads (Invitrogen, Waltham, MA, USA), the E4 mAb was purified following the manufacturer’s recommended protocol. Concentration was determined using a NanoDrop Spectrophotometer. Rounds of affinity selection were carried out on Immunlon II 96-well ELISA plates using a protocol previously described [24,26,34]. Wells were coated at 4 °C overnight with 250–500 ng of the purified E4 mAb in a total volume of 50 uL with PBS. Excess antibody was washed off five times with 150 μL of PBS. The wells were then blocked for 2 h with 0.5% (*w*/*v*) nonfat dry milk in PBS. Blocking solution was washed off five times with 150 μL of PBS. An MS2-VLP library displaying a random peptide library (6-, 7-, 8-, and 10-mers) was previously constructed [25]. Approximately 250 ng/well of the VLP random peptide library (6-, 7-, 8-, and 10-mers) in 0.5% (*w*/*v*) nonfat dry milk in PBS was added to the wells and incubated at room temperature for 2 h. Control wells were incubated with 0.5% (*w*/*v*) nonfat dry milk in PBS with no VLPs. The wells were washed six times with 150 μL of PBS. The VLPs were then eluted by addition of 50 μL of a 0.1 M glycine pH 2.7, followed by 5–10 min incubation at room temperature. After removal of VLPs, immediate neutralization took place by adding 5 μL of 1 M Tris, pH 9.0. The RNeasy Micro kit (Qiagen, Germantown, MD, USA) was used to purify encapsidated RNA from the eluted VLPs, following the manufacturer’s protocol.

### 2.2. RT-PCR of Affinity Selected VLPs and Confirmation of PCR Product

RT-PCR was performed in order to obtain cDNA derived from the VLP-associated RNA, following a previously described protocol [28,29,30,31,32]. Briefly, for the RT reaction, each reaction mixture contained 4 μL of 10 μmol/L E2 primer (5′–TCAGCGGTGGCAGCAGCCAA–3′), 1 μL of 10 mmol/L dNTP mix (Invitrogen, Waltham, MA, USA), 4 μL of 5× First Strand Buffer (Invitrogen, Waltham, MA, USA), 2 μL of 0.1 mol/L DTT (Invitrogen), and 1 μL of MMLV-RT (Invitrogen, Waltham, MA, USA). Primers, dNTPs, and 8 μL of extracted RNA were mixed and placed at 65 °C for 5 min. The samples were removed and placed on ice for a quick chill. Then, 5× First Strand Buffer and 0.1 M DTT were added, and the sample tubes were heated to 37 °C for 2 min. Next, 1 μL of MMLV-RT (Invitrogen, Waltham, MA, USA) was added and mixed. The thermocycler ran for 50 min at 37 °C and 15 min at 70 °C, before holding at 4 °C. Primers for PCR amplification were E3.2 (5′–CGGGCTTTGTTAGCAGCCGG–3′) and 62 up (5′–CTATGCAGGGGTTGTTGAAG–3′). Here, 49 μL of PCR reaction mixture was mixed with 1 μL of the RT reaction. PCR settings were as follows: 94 °C for 2 min, 30 cycles of 94 °C for 30 s, 60 °C for 30 s, and 68 °C for 30 s, followed by a final extension at 68 °C for 10 min, before holding at 4 °C. To confirm PCR amplification, 8 μL of PCR product was mixed with 2 μL of 10× Orange G loading dye and assessed on a 1% agarose gel made with ethidium bromide, run at 90 V for 30 min. PCR products were purified with a Qiagen kit (Qiagen, Germantown, MD, USA) according to the manufacturer’s protocol. The DNA was eluted with 50 μL of the manufacturer’s provided Buffer EB.

### 2.3. Digestion of Purified PCR Products and Gel Purification

The purified PCR products were digested with a reaction mixture containing 5 μL of CutSmart Buffer (New England Biolabs, Ipswich, MA, USA), 2.5 μL of BamHI-HF, 2.5 μL of SalI-HF, and 12 μL of ddH_2_O. PCR product (28 μL) was added to the reaction mixtures and incubated for 1 h at 37 °C. The digests were assessed on a 1% agarose gel made with ethidium bromide, run at 90 V for 45 min. The digested PCR product was isolated using the Qiagen Gel Extraction kit (Qiagen, Germantown, MD, USA). DNA was eluted with 50 μL of the manufacturer’s provided Buffer EB.

### 2.4. Ligation and Transformation

Ligation of PCR product to the pDSP62 plasmid and transformation of *E. coli* were carried out according to a previously published protocol [30,31]. Briefly, each ligation mixture contained 10 μL of PCR digest, 2 μL of vector digest, 2 μL of 10× Ligation Buffer, 5 μL of ddH_2_O, and 1 μL of T4 DNA ligase. A digested vector pDSP62 was used [25]. Ligation mixtures were incubated at 4 °C overnight. The reaction was heated at 70 °C for 10 min to inactivate the enzyme. The reaction was then cleaned up with ethanol precipitation, resuspending the DNA pellet in 20 μL of ddH_2_O.

In order to transform *E. coli* with the ligation reactions, 150 μL (~3 × 10^9^ competent cells) of 10 G *E. coli* cells (Lucigen, Middleton, WI, USA) were added directly to each purified ligation, gently mixed by flicking the tube, and then incubated on ice for ~5 min. This was then divided into two electroporation cuvettes (~80 μL total) shocked on the EC1 setting (Bio-Rad Electroporator, Bio-Rad, Hercules, CA, USA). Cells were transferred into 100 mL of LB media for recovery at 37 °C with shaking for 1 h. After recovery, kanamycin was added (50 μg/mL) and incubated overnight at 37 °C with shaking. Plasmids were subsequently isolated from these cultures using the QIAFilter Plasmid Midi Kit following the manufacturer’s protocol (Qiagen, Germantown, MD, USA. The number of total transformants was calculated by plating 10-fold serial dilutions of the culture after recovery on LB agar plates with kanamycin.

### 2.5. Transformation of E. coli for VLP Expression

In order to generate new VLP selectant libraries after each round of selection, plasmid DNA from above was diluted 1:10, and 1 uL of this diluted plasmid DNA was added to 150 μL of C41 (DE3) cells. This was divided equally into two electroporation cuvettes (~75 μL) and electroporated on the EC1 setting (Bio-Rad Electroporator, Bio-Rad, Hercules, CA, USA). Transformed cells were then transferred to 250 mL of LB and allowed to recover at 37 °C for 1 h. Samples were taken to assess total transformants as described above. Kanamycin was then added (50 μg/mL), and the culture was allowed to grow at 37 °C with shaking until the OD at 600 nm was between 0.6 and 0.8. Recombinant coat protein expression was then induced by adding 0.4 mM IPTG. Cultures continued to shake at 37 °C for an additional 3 h to allow coat protein expression and VLP formation. Bacterial pellets were obtained by centrifugation of the culture at 3750 rpm for 30 min at 4 °C.

### 2.6. Isolation of VLPs

Lysis of bacterial cultures was carried out to obtain purified VLPs. Cell pellets were resuspended in 5 mL of lysis buffer (10 mL of NaCl, 10 mL of 0.5 M EDTA, and 25 mL of 1 M Tris pH 8.5 in 500 mL of ddH_2_O) and incubated on ice for 1 h. Next, 25 μL of 10% deoxycholate was added to the suspension and incubated on ice for 30 min. Samples were then sonicated three times for 30 s pulses. MgCl_2_ (20 mM final concentration) and DNase (20 μg/mL) were then added and incubated at room temperature for 1 h to remove DNA. Insoluble material was then removed by centrifugation at 3750 rpm for 30 min at 4 °C. Supernatants containing soluble VLPs were saved (crude VLPs). The presence of VLPs was confirmed by assessing 25 μL of crude VLPs mixed with 5 μL of gel loading dye on a 1% agarose gel stained with ethidium bromide, run at 90 V for 40 min. Wildtype MS2 VLPs were used as a positive control for gel electrophoresis. These crude VLP preparations were then used for the next round of affinity selection, repeating the protocol from Section 2.1, Section 2.2, Section 2.3, Section 2.4, Section 2.5 and Section 2.6 three times for four total rounds of affinity selection.

### 2.7. PCR with Barcode Primers, Digests, and Gel Extraction for Ion Torrent Deep Sequencing

Each round of selection was assessed by deep sequencing to identify the population-level changes occurring. To prepare the samples for Ion Torrent deep sequencing, custom barcode primers were used to amplify the portion of the pDSP62 plasmid that contains the coding sequence of the foreign peptide. Contaminating wildtype plasmids were removed by digesting the plasmid DNA with KpnI restriction enzyme. Each enzyme digest contained 10 μg of plasmid DNA and H_2_O to 85 μL, 10 μL of 10× CutSmart, and 5 μL of KpnI-HF. This was incubated at 37 °C for 1 h. After the incubation, ethanol precipitation was carried out to remove the enzyme and concentrate the sample. The barcode PCR protocol was previously described [30]. Briefly, each PCR reaction mixture contained 50 μL of High-Fidelity Platinum Taq Polymerase (Invitrogen), and conditions were carried out according to the manufacture’s protocol. PCR settings were as follows: 94 °C for 2 min, 30 cycles of 94 °C for 30 s, 60 °C for 30 s, and 68 °C for 30 s, followed by 68 °C for 10 min, before holding at 4 °C. All 50 μL of the PCR sample was then mixed with 5 μL of gel loading dye and subjected to agarose gel electrophoresis on a 1.5% agarose gel made using TBE Buffer. Appropriate products were sliced from the gel using a UV transilluminator and extracted with a Gel Extraction kit (Qiagen, Germantown, MD, USA). DNA was eluted with 50 μL of Buffer EB. These samples were then sequenced by the University of New Mexico Comprehensive Cancer Center Analytical and Translational Genomics Shared Resource using the Ion Torrent next-generation sequencing technology. Sequencing data were analyzed as previously described [28,30,31,32].

### 2.8. Purification of VLPs Using Size-Column Chromatography

In order to purify VLPs of interest for immunizations, plasmids were isolated from individual bacterial colonies containing the plasmid of interest using Miniprep kits (Qiagen, Germantown, MD, USA). These plasmids were verified for appropriate sequences and then used to transform C41 cells. Plasmid DNA (0.01 μL) was mixed with 40 μL of C41 (DE3) cells and electroporated as described above. Cells were allowed to recover for 1 h and then plated on LB agar plates with kanamycin. One colony of each plate was inoculated into 4 mL of LB/kan and placed into the 37 °C shaker overnight. These inoculations were transferred into 100 mL of LB medium and shaken at 37 °C until the OD at 600 nm was between 0.6 and 0.8. Coat protein expression was induced with 0.4 mM IPTG for 3 h at 37 °C with shaking. Bacterial cell pellets were isolated by centrifugation at 3750 rpm for 30 min at 4 °C. Cell lysis and confirmation of VLPs took place as mentioned above. The crude VLP extracts were frozen–thawed and centrifuged at 10,000× *g* for 10 min to remove insoluble material. This was then fractionated by size exclusion chromatography over CL-40 Sepharose. Fractions containing VLPs were identified by agarose gel electrophoresis of fractions and SDS-PAGE. VLP-containing fractions were combined and concentrated using 100,000 MW cutoff Amicon Centrifuge Filter units according to manufacturer’s protocol, and the buffer was exchanged with PBS. VLP concentration was visually estimated by SDS-PAGE with Coomassie staining using known quantities of hen egg lysozyme as a standard.

### 2.9. Immunization and Challenge of Mice with MS2 and Purified VLPs

Animal work was carried out with the approval of the University of New Mexico HSC IACUC. BALB/c mice (five mice/group) were immunized with 5 μg of the VLP intramuscularly at 3 week intervals for a total of three doses so as to assure maximum antibody response [29]. A subset of mice (not utilized for challenge) underwent vaginal wash under inhaled isoflurane anesthesia and were sacrificed; blood was collected by cardiac puncture after a lethal dose of ketamine and xylazine. Vaginal washes were performed by inserting 30 μL of sterile PBS into the vaginal cavity and recovering the wash with pipetting. This was repeated for a total of three times per mouse. Sera were isolated by two sequential centrifugations of blood at 12,000 RPM for 30 min. Sera were stored at −20 °C until use. Mice undergoing vaginal challenge were administered 2.5 mg of medroxyprogesterone acetate (Amphastar Pharmaceuticals, Inc., Rancho Cucamonga, CA, USA) subcutaneously 7 days before challenge. Mice were administered 2 × 10^4^ IFU of luciferase-expressing *Chlamydia muridarum* (gift from Dr. Guangming Zhong, University of Texas Health San Antonio) vaginally in SPG [35]. *Chlamydia muridarum* is a commonly used surrogate infection model for investigating vaccines against *Chlamydia*, since it recapitulates many of the pathological aspects of urogenital *Chlamydia trachomatis* infection in humans and does not require instillation directly into the uterus to achieve reproducible ascended infection. Bacterial burden was determined using an in vivo imaging system (IVIS Spectrum, PerkinElmer, Waltham, MA, USA) on days 3 through 7 post infection, as previously described [35]. Mice were injected intraperitoneally with 0.2 mL of 40 mg/mL solution of D-luciferin in 1× PBS (Perkin Elmer). After 25 min, mice were positioned within the IVIS machine, and images were acquired for 1 min with the firefly probe. Average radiance was determined by selecting uniform regions of interest (ROIs) on the lower abdomen of each mouse. Control ROIs, placed on the chest cavity, were utilized to determine the background average radiance, which was subtracted from each experimental value. Analysis was completed using Live Image 4.3.1 software.

### 2.10. ELISA to Determine Antibody Titers

For the vaccinated mice sera dilution binding ELISA assay, a 96-well Immulon 2 plate (Thermo Scientific, Waltham, MA, USA) was coated with 0.5 μg/50 μL streptavidin (Invitrogen) in PBS at 4 °C overnight. Plates were washed three times with PBS. An SMPH crosslinker was then added at 1 μg/50 μL in PBS and incubated at room temperature for 1 h with rocking. Plates were then washed with PBS thrice, and then synthetic peptides (GenScript, Piscatawa, NJ, USA) were added at 1 μg/50 μL in PBS. After overnight incubation at 4 °C, plates were washed with PBS thrice and then blocked with 150 μL/well of 0.5% dry milk/PBS for 1 h. After two washes in PBS, serial dilutions of sera prepared in 0.5% milk/PBS were added and incubated for 2 h at room temperature. Plates were washed five times with PBS, and secondary antibody (HRP goat anti-mouse IgG, Jackson ImmunoResearch, West Grove, PA, USA) was added at 1:5000 in 0.5% milk/PBS. After incubation at room temperature for 45 min with rocking, plates were washed five times in PBS. 3,3′,5,5′-Tetramethylbenzidine (TMB) substrate (EMD Millipore Corp, Burlington, MA, USA) was added and allowed to react until sufficient color developed to read. Reactions were quenched with 50 μL of 1% HCl and read with a Fisher Scientific accuSkan FC plate reader at the setting 450 nm TMB.

For the monoclonal antibody dilution binding ELISA assay, a 96-well Immulon 2 plate (Thermo Scientific, Waltham, MA, USA) was coated with 250 ng/50 μL of MS2-HMVGSTKWTN and MS2 VLPs in PBS in respective wells at 4 °C overnight. Plates were washed three times with PBS, and 50 μL of 0.5% nonfat dry milk/PBS was then added. After overnight incubation at 4 °C, the plate was washed with PBS thrice, and then 50 μL of fourfold dilutions of the E4 mAb in 0.5% milk in PBS, starting at 1:20, were added to the respective wells. The plate was then incubated for 2.5 h at room temperature with gentle rocking. The wells were washed five times with PBS, and secondary antibody (HRP goat anti-mouse IgG-Jackson, ImmunoResearch, West Grove, PA, USA) was added at 1:5000 in 0.5% milk/PBS. After incubation at room temperature for 1 h with gentle rocking, the plate was washed three times in PBS. TMB substrate (EMD Millipore Corp, Burlington, MA, USA) was added and allowed to react until sufficient color developed to read. Reactions were quenched with 50 μL of 1% HCl and read with a Fisher Scientific accuSkan FC plate reader at the setting 450 nm TMB.

The competition ELISA was performed as a peptide ELISA utilizing a synthetic peptide corresponding to the Ct serovars D and E VD4 region with a short linker sequence (FDTTTLNPTIAGAGDVK-GGGC, subsequently referred to as the CtsvDE VD4 peptide), as above with some differences. The murine E4 mAb was purified by Protein G Dynabeads (Invitrogen) according to manufacturer’s instructions and diluted to 1:160 in PBS-0.5% dry milk. The E4 mAb was then mixed with various competitors, including the CtsvDE VD4 peptide (final concentration 0.5 μg/μL), MS2-WT VLP (final concentration 125 ng/μL), or MS2-HMVGSTKWTN (final concentration 125 ng/μL). Each competitor underwent fourfold dilutions, allowing for decreasing amounts of competitor to be mixed with the same concentration of E4 mAb. The E4 mAb was mixed with each competitor or with PBS–0.5% dry milk (control). Then, 100 μL volumes were added to the peptide ELISA (in place of immune sera), followed by HRP goat anti-mouse IgG, TMB (five minutes), and 1% HCl, with appropriate washes in between.

### 2.11. Elementary Body ELISA

EB ELISA was performed as previously described [36]. CtsvD EB lysate total protein concentration was determined using a bicinchoninic acid (BCA) assay (Pierce), as per the manufacturer’s protocol. All washes were completed three times in 0.05% PBS–Tween-20 (PBST), and incubations were completed at room temperature, unless otherwise noted. Briefly, Immulon 2 HB 96-well flat-bottom microtiter plates (Thermo Scientific, Waltham, MA, USA) were coated with 75 μL of 1 μg/mL poly-L-lysine diluted in 0.05 M bicarbonate buffer (pH 9.6) and incubated overnight at 4 °C. After washing plates, 50 μL of CtsvD EBs were added to each well in PBS at a final concentration of 4.5 μg/mL. Plates were centrifuged at 900× *g* for 5 min and fixed with 50 μL of 0.1% glutaraldehyde in PBS for 20 min. Plates were again washed, and 300 μL of 2% goat serum-PBST (blocking agent) was added to each well and incubated overnight at 4 °C. Immune sera was serially diluted in blocking agent, and 100 μL was added to each well; incubation with rocking occurred for 2 h. Plates were washed four times and reacted with 1:5000 peroxidase-conjugated anti-mouse IgG secondary (Jackson ImmunoResearch, West Grove, PA, USA) for 30 min with rocking. Plates were washed four times and incubated with 100 μL 3,3′,5,5′-tetramethylbenzidine (TMB, EMD Millipore, Burlington, MA, USA) for 20 min before being quenched with 1% hydrochloric acid. Absorbance was read at 450 nm using accuSkan FC (Fisher Scientific, Waltham, MA, USA). The background was averaged and subtracted from each experimental and control well.

### 2.12. In Vivo Neutralization Assay

Naïve female Balb/c mice (7 weeks of age, *n* = 10/group, Jackson Labs, Bar Harbor, ME, USA) were administered 2.5 mg of medroxyprogesterone acetate 7 days prior to infection. A 1:1 volume mixture of previously collected mixed terminal immune sera and luciferase-expressing Cm in a total volume of 20 μL was utilized for vaginal challenge. Mixed terminal immune sera were heat-inactivated for 30 min at 56 °C. Terminal immune sera were mixed with Luc-Cm to achieve a final concentration of IFU of 2 × 10^4^ in 20 μL mixed volumes. Mixture was incubated at 37 °C for 30 min with occasional mixing. Mice were administered their respective mixtures, and IVIS was performed as described above.

## 3. Results

### 3.1. Conformational Mimics of the E4 Epitope Identified Utilizing Affinity Selection

**Figure 1:**
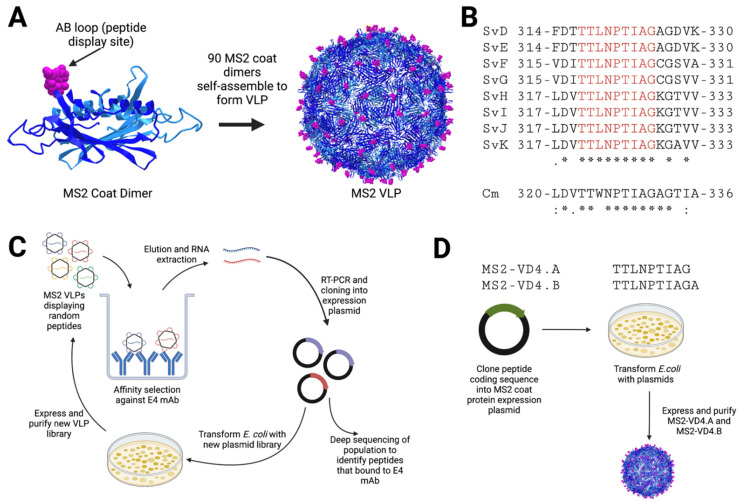
Schematic of MS2 bacteriophage VLP-based approaches for MOMP VD4 conserved epitope vaccine design. (**A**) MS2 bacteriophage coat protein dimers each display one foreign peptide in the surface-exposed beta-hairpin, called the AB loop. (**B**) The sequence of the VD4 core conserved epitope (TTLNPTIAG) is shown in alignment for all urogenital Ct serovars (* perfect conservation), as well as Cm. Cm alignment scores are as compared to CtsvD (shown below Cm sequence), and alignment scores of all urogenital serovars are shown below Ct SvK. (**C**) Affinity selection approach against E4 mAb. An MS2 VLP library displaying random peptides in the AB loop is panned against the E4 mAb. Bound VLPs are eluted, and encapsidated coding RNA is extracted. This is then subjected to RT-PCR and cloning into the MS2 VLP expression plasmid, which is then transformed into *E. coli* to produce a new VLP library that is enriched for VLPs that bind to E4 mAb. The process is then repeated for four total rounds. The RT-PCR product from the final round is then subjected to deep sequencing to identify peptides of interest that bind to E4 mAb. (**D**) Rational-design approach using display of the VD4 core conserved epitope MS2 VLPs. The coding sequences for peptides are cloned into an MS2 coat protein expression plasmid such that the peptide is displayed in the AB loop. Plasmids are then transformed into *E. coli*, and VLPs are expressed and purified.

As an initial vaccination strategy, we used affinity selection to identify VLP-based immunogens displaying conformational mimics of the E4 epitope. Starting with a random peptide library displayed on the surface of bacteriophage MS2 VLPs [25], we performed four rounds of affinity selection (Figure 1C). Following the final round of selection, selected clones were subjected to deep sequencing; roughly 55% of the total selectants displayed the peptide sequence HMVGSTKWTN (Table 1). This peptide had no sequence similarity to the VD4 MOMP conserved region or any portion of the Ct MOMP protein as determined by NCBI BLAST, suggesting that it may be a conformational mimic of the E4 epitope. Several other selectants with sequence similarities were also identified (shown in bold, Table 1). The second most common selectant was GVFYGSS, accounting for 21.51% of selectants.

### 3.2. E4 mAb Binds to MS2-HWVGSTKWTN VLPs but Not to a Synthetic HMVGSTKWTN Peptide

**Figure 2:**
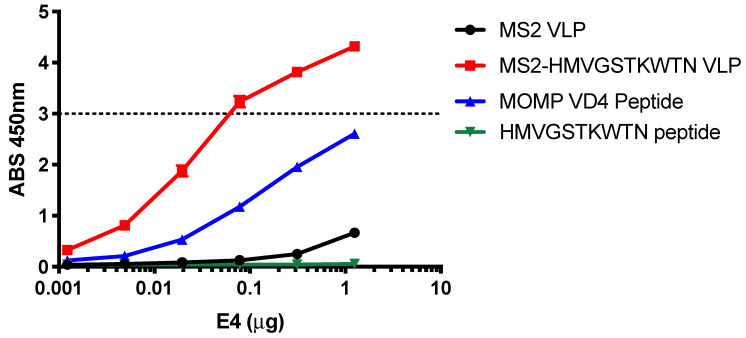
E4 mAb binds to HMVGSTKWTN peptide in a conformation-dependent manner. E4 mAb binding to linear HMVGSTKWTN peptide, MS2-HMVGSTKWTN (MS2 VLP displaying HMVGSTKWTN in the AB loop), MS2 VLP (negative control), and VD4 epitope linear peptide (FDTTTLNPTIAGAGDVK) was assessed by ELISA at various concentrations of E4 mAb. Data are the average of three technical replicates. The dashed line represents the linear limit of the ELISA plate reader.

After identifying the HMVGSTKWTN peptide as a potential conformational mimic of the E4 mAb epitope with our affinity selection platform, we next characterized the binding of the E4 mAb against this peptide by ELISA. E4 mAb binding to the HMVGSTKWTN peptide alone (linear peptide), MS2-HMVGSTKWTN VLP (peptide displayed in constrained AB loop of MS2 coat protein), MS2 VLP (no peptide displayed, control), and MOMP VD4 peptide (FDTTTLNPTIAGAGDVK) was measured (Figure 2). Similar to data previously reported [10], the E4 mAb bound to the MOMP VD4 peptide containing the LNPTIAG conserved epitope with flanking amino acids (Figure 2). We also observed binding of the E4 mAb to the MS2-HMVGSTKWTN VLP, but not the MS2 VLP control or the linear HMVGSTKWTN peptide. These results indicate that displaying the HMVGSTKWTN peptide in a constrained loop on the surface of the MS2 VLP is critical for E4 mAb binding.

### 3.3. Mice Immunized with MS2-HMVGSTKWTN VLP Produce Antibodies That Bind to Chlamydia EBs

**Figure 3:**
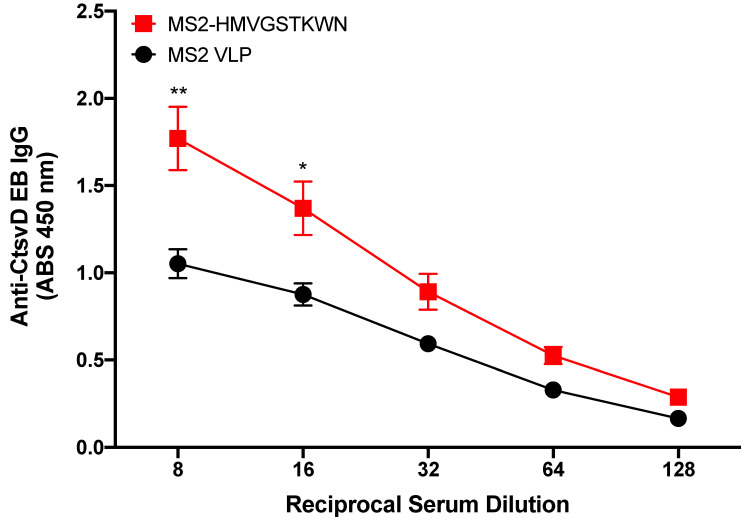
Binding characteristics of MS2-HMVGSTKWTN immunized mouse sera IgG to Ct serovar D. Mice (*n* = 5) were immunized with MS2-HMVGSTKWTN or WT MS2 (negative control). Sera were collected, and binding activity was assessed against Ct serovar D by EB ELISA. Statistical analysis was performed utilizing a nonparametric Mann–Whitney *t*-test. Quantitative data represent the mean ± SEM. * *p* < 0.05, ** *p* < 0.01.

We next hypothesized that the MS2-HMVGSTKWN VLP would elicit antibodies that could bind to Ct EBs. In order to test this, we immunized mice with MS2-HMVGSTKWN VLPs and tested the sera IgG for binding to Ct serovar D EBs by ELISA (Figure 3). Immune sera IgG from experimental mice showed statistically significant higher binding to EBs than control immune sera at sera dilutions of 1:8 and 1:16, but not at higher dilutions, indicating that there was only weak binding of immune sera to EBs.

### 3.4. Preferential Binding of E4 mAb to the VD4 Epitope over the Conformational Mimic

**Figure 4:**
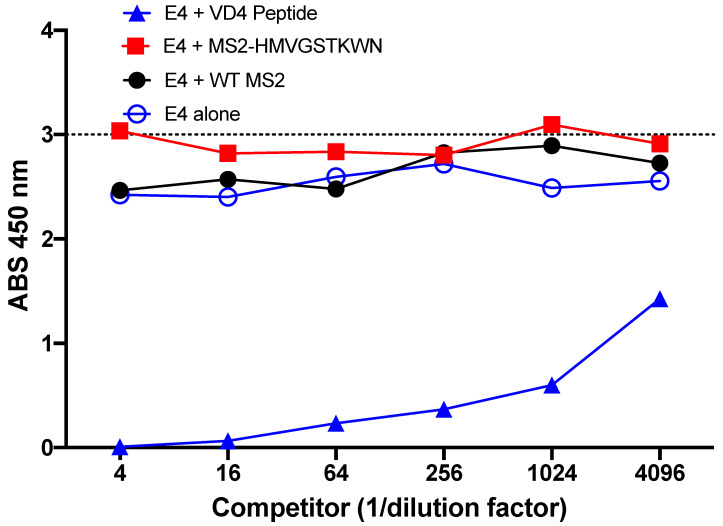
Preferential binding of E4 mAb. Competition peptide ELISA revealed that addition of MS2-HMVGSTKWN as a binding competitor of E4 mAb did not change the binding potential to the VD4 peptide, whereas addition of the linear VD4 peptide reduced binding of the E4 mAb to the bait VD4 peptide.

Next, we performed a competition ELISA to determine if E4 mAb preferred binding to the core epitope or the conformational mimic. In this experiment, a VD4 peptide was used as bait, and then E4 mAb was added alone (E4 alone) or in the presence of different amounts of potential competitors (linear VD4 peptide or MS2-HMVGSTKWTN) or WT MS2, as a negative control. We found that addition of WT MS2 had little impact on E4 mAb binding to the VD4 peptide, as expected, with similar absorbance values to E4 mAb alone. Likewise, addition of MS2-HMVGSTKWTN had little effect on the binding of E4 mAb. However, when the VD4 linear peptide was added as the competitor, absorbance values were dramatically reduced (Figure 4). Together, this demonstrates that the E4 mAb preferentially binds to the VD4 peptide over the affinity-selected conformational mimic, MS2-HMVGSTKWTN.

### 3.5. E4 mAb Binds to MS2 VLPs Displaying VD4 Conserved Epitope

**Figure 5:**
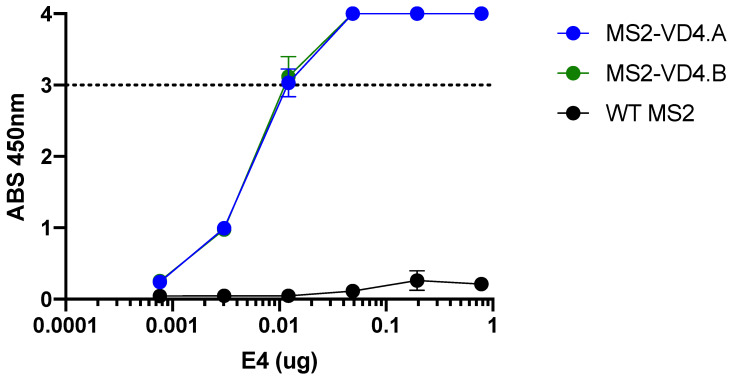
E4 mAb binds to MS2 VLPs displaying the VD4 epitope. E4 mAb binding capacity to MS2-VD4.A and MS2-VD4.B as measured by ELISA, demonstrating binding above that of WT MS2 across a range of dilutions. Quantitative data represent the mean ± SD.

Next, we engineered MS2 VLPs displaying two related versions of the VD4 epitope (MS2-VD4.A, TTLNPTIAG; MS2-VD4.B, TTLNPTIAGA) within the AB loop of the coat protein. The E4 mAb bound to both MS2-VD4.A and MS2-VD4.B across a range of dilutions (Figure 5). This suggests that, when displayed on the surface of MS2 VLPs in a constrained loop, the core conserved VD4 peptide epitope takes on a conformation relevant for E4 mAb binding.

### 3.6. Immunization with MS2 VLPs Displaying the VD4 Epitope Results in High-Titer, Specific Serum IgG Antibodies

**Figure 6:**
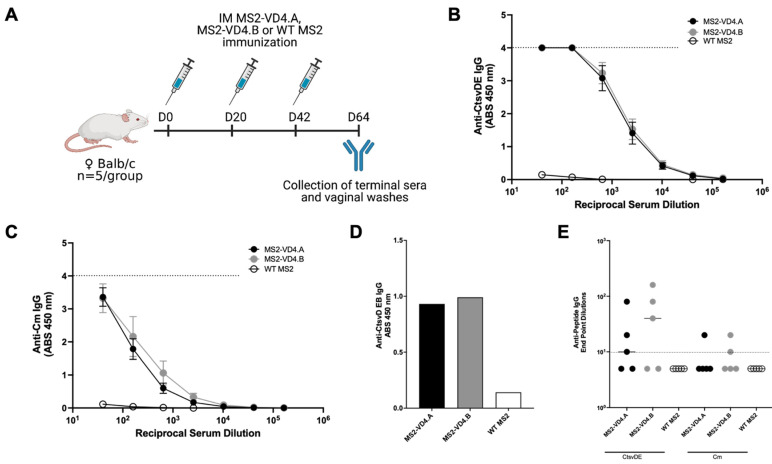
MS2-VD4 immunization results in high-titer, epitope-specific IgG antibody responses. (**A**) Immunization schedule. Female Balb/c mice (*n* = 5/group) were immunized three times, 3 weeks apart with their respective vaccine before collection of terminal immune sera and vaginal washes to investigate antibody responses via ELISA. (**B**) Binding capacity of MS2-VD4 immune sera IgG to the CtsvDE VD4 epitope peptide (FDTTTLNPTIAGAGDVK). (**C**) Binding capacity of MS2-VD4 immune sera IgG to the Cm VD4 epitope peptide (LDVTTWNPTIAGAGTIA). (**D**) Binding capacity of MS2-VD4 immune sera IgG to CtsvD EBs by ELISA. Serum dilution 1:128. (**E**) Binding capacity of mucosal IgG from vaginal washes to CtsvDE (left) and Cm (right) VD4 epitope peptide. Quantitative data represent the mean ± SEM (**B**,**C**) and median (**E**).

Next, we investigated the antibody response elicited by immunization with MS2-VD4 VLPs. We vaccinated female Balb/c mice with MS2-VD4.A, MS2-VD4.B, or WT MS2 three times intramuscularly, so as to assure maximum antibody response to the antigen. Vaginal washes and serum were collected approximately 3 weeks after the final immunization to assess antibody responses (Figure 6A). Both MS2-VD4.A and MS2-VD4.B elicited IgG that bound strongly to the CtsvDE VD4 peptide epitope (FDTTTLNPTIAGAGDVK-GGGC, Figure 6B). IgG from vaccinated mice also bound to synthetic peptides representing the VD4 epitopes from a panel urogenital Ct serovars; these epitopes have the same core epitope but variable flanking amino-acid sequences (Supplementary Figure S1). We next investigated if immune sera IgG could bind to CtsvD EBs. Due to low volumes of serum available for testing, we pooled murine immune sera and performed an ELISA against CtsvD EBs using a single (1:128) dilution. Pooled immune sera IgG bound to CtsvD EBs at a higher absorbance value than MS2 WT immune sera IgG (Figure 6D). We also investigated if epitope-specific IgG was present within the vaginal tract of immunized mice and found that a subset of mice had detectable antibody responses at this mucosal site (Figure 6E).

### 3.7. Mice Immunized Intramuscularly with MS2-VD4.A Are Protected against Vaginal Chlamydia Infection

**Figure 7:**
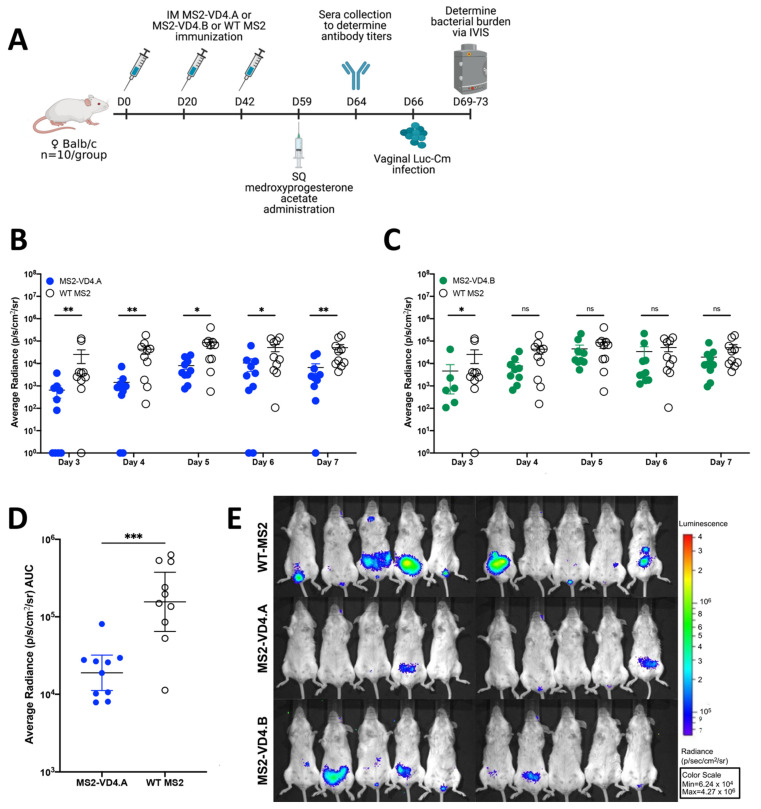
Immunization with MS2-VD4.A results in decreased urogenital *Chlamydia* burden as compared to WT MS2 immunized controls. (**A**) Immunization schedule. Female Balb/c mice (*n* = 10/group) were immunized three times, 3 weeks apart with their respective vaccine. Mice were administered 2.5 mg of Depo-Provera before vaginal challenge with Luc-Cm. (**B**) Average radiance (p/s/cm^2^/sr) measured for MS2-VD4.A and WT MS2 on days 3 through 7 post infection. (**C**) Average radiance (p/s/cm^2^/sr) measured for MS2-VD4.B and WT MS2 on days 3 through 7 post infection. (**D**) Area under the curve (AUC) measured for average radiance on days 3 through 7 post infection for MS2-VD4.A and WT MS2. (**E**) IVIS images for WT MS2 control mice (top), MS2-VD4.A mice (middle), and MS2-VD4.B mice (bottom) on day 7 post infection, with luminescence visualized, shown as a representative image of those collected each day. Statistical analysis was performed utilizing the nonparametric Mann–Whitney *t*-test. Quantitative data represent the geometric mean ± SEM. * *p* < 0.05, ** *p* < 0.01, *** *p* < 0.001.

Next, we tested the ability of MS2-VD4 vaccines to prevent *Chlamydia* infection in a female mouse urogenital challenge model. Cm is closely related to Ct and is a well-described model organism for assessing urogenital *Chlamydia* infection in mice [37,38]. Cm challenge in the female urogenital tract of mice has been used extensively to assess vaccine candidates and study the immune response and pathology of urogenital *Chlamydia* infection. We chose to first assess our vaccines in the Cm female vaginal challenge model because Cm recapitulates many of the early aspects of human female reproductive tract infection, including lower genital tract exposure and infection of the cervix prior to ascension into the upper reproductive tract. However, amino-acid differences in MOMP between Cm and Ct require additional considerations. To determine whether our vaccines could be assessed in the female urogenital Cm model, we investigated whether MS2-VD4 immune sera could bind to Cm. Indeed, MS2-VD4 immune sera IgG bound to the Cm homolog of the conserved VD4 epitope despite amino-acid sequence differences (Figure 6C). Female Balb/c mice were immunized three times with MS2-VD4 vaccines and then vaginally challenged with luciferase-expressing Cm (Luc-Cm) [35]. Luc-Cm (2 × 10^4^ IFU) was administered vaginally, and bacterial burden, measured by average radiance, was monitored via in vivo imaging (IVIS) days 3 through 7 post infection (Figure 7A). Immunization with MS2-VD4.A resulted in decreased bacterial burden over all days investigated (Figure 7B,D). Mice immunized with MS2-VD4.A had a 1.02 log decrease in mean bacterial burden over the course of infection compared to control mice immunized with WT MS2 (Figure 7D). However, immunization with MS2-VD4.B did not lead to a similar decrease in bacterial burden. Although mean bacterial burden in this group was lower than controls throughout infection, this difference was only statistically significant at day 3 post infection (Figure 7C). The decrease in bacterial burden in mice vaccinated with MS2-VD4.A can be visualized in an IVIS image taken at day 7 post infection (Figure 7E).

### 3.8. Protection against Primary Vaginal Chlamydia Infection Can Be Mediated by MS2-VD4.A Antibodies

**Figure 8:**
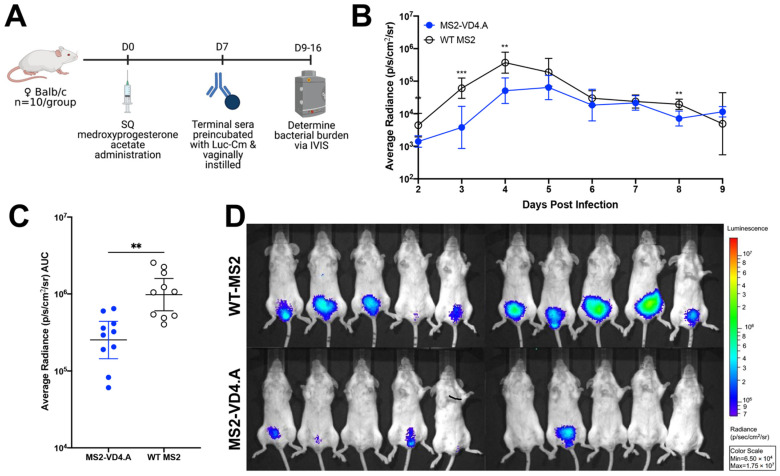
Protection from vaginal *Chlamydia* infection can be mediated by antibodies. (**A**) Study design for in vivo neutralization. Naïve female Balb/c mice (*n* = 10/group) were vaginally challenged with 2 × 10^4^ IFU of Luc-Cm that was preincubated with terminal immune sera from mice vaccinated with either MS2-VD4.A or WT MS2. Bacterial burden was determined on days 2 through 9 post infection. (**B**) Average radiance (p/s/cm^2^/sr) measured for mice receiving Luc-Cm preincubated with either MS2-VD4.A or WT MS2 immune sera on days 2 through 9 post infection. (**C**) Area under the curve (AUC) measured for average radiance on days 2 through 9 post infection. (**D**) IVIS image for mice receiving Luc-Cm preincubated with either WT MS2 immune sera (top) or MS2-VD4.A immune sera (bottom) on day 3 post infection, with luminescence visualized, shown as a representative image of those collected each day. Statistical analysis was performed utilizing the nonparametric Mann–Whitney *t*-test. Quantitative data represent the geometric mean ± SEM. ** *p* < 0.01, *** *p* < 0.001.

We next investigated if protection afforded by MS2-VD4.A was mediated primarily by antibodies by performing an in vivo neutralization assay. Luc-Cm was preincubated with immune serum from mice vaccinated with MS2-VD4.A or, as a negative control, WT MS2, and then was administered vaginally to naïve female Balb/c mice. Bacterial burden was measured daily on days 2 through 9 post infection (Figure 8A) using IVIS. Mice receiving the Luc-Cm preincubated with MS2-VD4.A had statistically lower bacterial burden at early timepoints post infection (Figure 8B). There was a 0.58 log decrease in mean bacterial burden over the days investigated relative to the control group (Figure 8C). Lower bacterial burden can be visualized by luminescence on IVIS images day 3 post infection (Figure 8D).

## 4. Discussion

Here, we described our efforts to target the Ct core conserved MOMP-VD4 epitope, TTLNPTIAG, using bacteriophage VLP-based vaccination approaches. We utilized two approaches to vaccine design. First, we performed affinity selections of bacteriophage MS2 VLPs displaying a library of random peptides (6-, 7-, 8-, and 10-mers) using an anti-MOMP mAb that neutralizes all urogenital Ct serovars. This approach identified a VLP displaying the peptide HMVGSTKWTN as a potential conformational mimic of the E4 mAb epitope. This was supported by the strong binding of E4 mAb to the MS2-HMVGSTWKN VLP but not the HMVGSTWKN peptide alone and the binding of sera from MS2-HMVGSTWKN immunized mice to Ct EBs. However, a competition ELISA revealed that the E4 mAb preferentially bound to the VD4 epitope, rather than the conformational epitope. As an alternative approach, we engineered VLPs in which the TTLNPTIAG(A) epitope was displayed in the AB loop of the MS2 coat protein. These VLPs, which were also recognized by E4 mAb, elicited anti-peptide serum IgG antibodies at ~10^4^ endpoint dilution titer after three immunizations and, in a subset of vaccinated mice, detectable IgG in the genital tract. Importantly, IgG from MS2-VD4 vaccinated mice bound to Ct EBs, indicating that these antibodies could bind to native MOMP. We did not detect peptide-specific IgG in the vaginal tract of all vaccinated mice. One possibility for this variation between mice is that IgG in the vaginal tract fluctuates over time according to the estrous cycle, and our mice did not have synced estrous cycles. Additionally, the amount of peptide-specific IgG that we detected in the mouse vagina was quite low; thus, it is possible that there was peptide-specific IgG but that it was below our limit of detection.

Although MS2-VD4.A and MS2-VD4.B differed only by a single C-terminal alanine, MS2-VD4.A vaccination resulted in better protection against a vaginal *Chlamydia* challenge in mice. This is supported by the fact that a single amino-acid addition or deletion in the peptide on MS2 led to differences in bacterial burden in immunized mice. These data suggest that protection afforded by antibodies against the VD4 conserved epitope may be dependent on subtle conformational aspects of the core conserved peptide. There are currently no published crystal or NMR structures of Ct MOMP protein, limiting our ability to determine with confidence the conformation of the VD4 region to aid in vaccine design. However, several protein structure prediction models have been generated over the years, pointing to a trimeric beta-barrel structure with surface-exposed loops corresponding to the variable domains [39]. A high-confidence predicted protein structure of MOMP has been generated using AlphaFold (AF-Q46409-F1, [34,40], Supplementary Figure S2). This AlphaFold model shows that the VD4 region and the VD4 conserved epitope TTLNPTIAG, in particular, have low model confidence scores. This suggests that the conformation of this region may vary during the course of infection. This is supported by previous structural modeling and experimental evidence for various *Chlamydia* species MOMP proteins that suggest that the conserved VD4 epitope TTLNPTIAG may be variably exposed, depending on the environmental conditions, and they are likely involved in porin function, explaining the high conservation of this region in an otherwise variable region [39,41]. The VD4 conserved epitope, TTLNPTIAG, is located in an extended loop structure and appears to be unconstrained in the AlphaFold model (Supplementary Figure S2). Together, this information along with our data suggest that vaccines that elicit antibodies to the MOMP VD4 conserved TTLNPTIAG epitope may benefit from optimization to elicit antibodies to specific constrained conformations of the epitope.

Here, we present a novel approach to Ct vaccine development by using the bacteriophage MS2 VLP to target the conserved epitope of VD4 MOMP. Although intramuscular immunization using MS2-VD4.A elicited high titer serum IgG and provided protection from genital challenge, it is possible that alternative vaccination strategies, including approaches that incorporate adjuvant or mucosal delivery, could provide better protection from infection. One advantage of targeting the VD4 region is that this core epitope is relatively conserved amongst urogenital Ct serovars, and we demonstrated that MS2-VD4.A elicited antibodies that bound synthetic peptides representing these different serovars. Although our animal model choice limited our ability to test binding to additional Ct EB serovars, we hypothesize that our vaccine approach may be able to provide cross-protection due to the conserved nature of the target epitope. The immune sera IgG also bound the Cm peptide epitope, albeit to a lesser extent. Therefore, potential protection against Ct serovars may be even higher than the 1.02 log decrease in mean bacterial burden seen with Luc-Cm challenge.

## 5. Conclusions

Immunization with MS2-VD4.A was able to elicit antibodies at >10^4^ endpoint dilution titer that were found both peripherally and within the vaginal mucosa and resulted in protection against a vaginal *Chlamydia* challenge in mice. Future studies will investigate this vaccine in other relevant *Chlamydia* challenge models.

## Figures and Tables

**Table 1:** The top 12 peptides identified by affinity selection.

Peptide Sequence	% of Total VLPs Displaying Peptide Sequence
H**MVG**S**TKWT**N ^1^	54.77
GVFYGSS	21.51
GAFYRHS	11.35
EVWGVGP	3.07
SSFYGSD	0.76
WGTRHSP	061
H**MVG**P**TKWT**N ^1^	0.37
TNGWGP	0.24
GKWVGGLGTA	0.20
HMVGSTKWTS	0.20
GAFYQHS	0.18
R**MVG**S**TKWT**N ^1^	0.14

^1^ Amino acids in boldface are shared among the XMVGXTKWTX family of peptides.

## Data Availability

The data presented in this study are available in the article.

## Supplementary Materials

The following supporting information can be downloaded at https://www.mdpi.com/article/10.3390/vaccines10060875/s1: Figure S1. Immunization with MS2-VD4 results in high-titer IgG antibodies that recognize all Ct serovars; Figure S2. AlphaFold model of Ct serovar D MOMP protein structure.
