# Supplementary material for: Epitope-Based Vaccines against the Chlamydia trachomatis Major Outer Membrane Protein Variable Domain 4 Elicit Protection in Mice
Source: Vaccines (Basel). 2022 May 30;10(6):875. doi: 10.3390/vaccines10060875 (PMC9227494; doi:10.3390/vaccines10060875)
Supplement: Supplementary file 1 [file vaccines-10-00875-s001.zip › vaccines-1726379-SI.pdf]

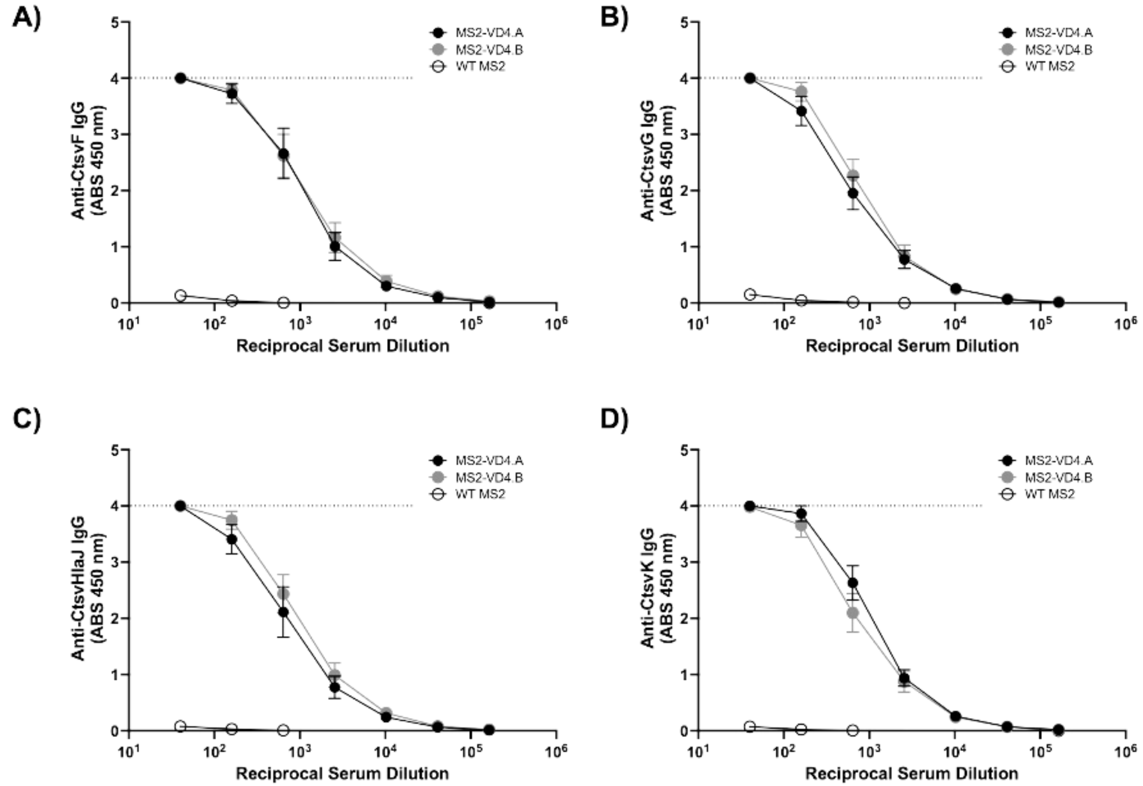

Supplementary Figure S1. MS2-VD4.A and MS2-VD4.B immunized mouse sera binding to VD4 peptides from urogenital Ct serovars. Sera from mice immunized 3 times with MS2-VD4.A, MS2-VD4.B, or WT MS2 was tested by ELISA for binding to peptides representing the VD4 region of various urogenital Ct serovars. Peptide sequences are shown in Figure 1.

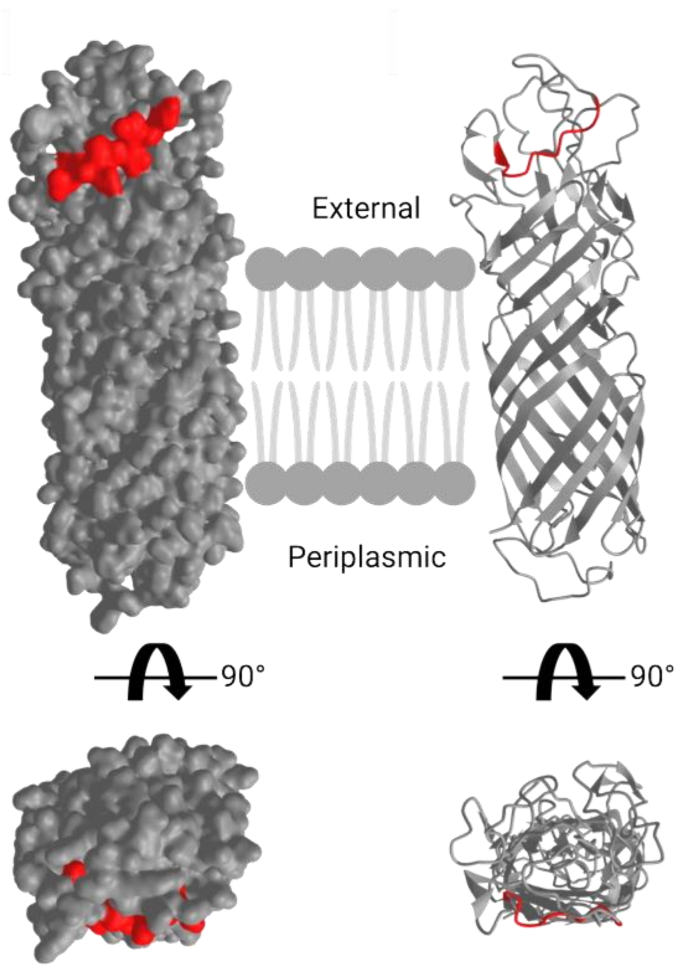

Supplementary Figure S2. AlphaFold model of Ct serovar D MOMP protein structure. AlphaFold structural model of Ct serovar D MOMP (AF-Q46409-F1) was used to generate the above images in iCn3D (Wang J, Youkharibache P, Zhang D, Lanczycki CJ, Geer RC, Madej T, Phan L, Ward M, Lu S, Marchler GH, Wang Y, Bryant SH, Geer LY, Marchler-Bauer A. iCn3D, a Web-based 3D Viewer for Sharing 1D/2D/3D Representations of Biomolecular Structures. ***Bioinformatics.*** **2020** Jan 1;36(1):131-135. (Epub 2019 June 20.) doi: 10.1093/bioinformatics/btz502). Red residues highlight the conserved TTLNPTIAG VD4 epitope.
